# Gene expression regulation in the context of mouse interspecific mosaic genomes

**DOI:** 10.1186/gb-2008-9-8-r133

**Published:** 2008-08-27

**Authors:** David L'Hôte, Catherine Serres, Reiner A Veitia, Xavier Montagutelli, Ahmad Oulmouden, Daniel Vaiman

**Affiliations:** 1 U567 Department of Genetics and Development, Institut Cochin, INSERM, 24 rue du Faubourg St Jacques, Paris, 75014, France; 2 UMR8104 Department of Genetics and Development, Institut Cochin, CNRS, 24 rue du Faubourg St Jacques, Paris, 75014, France; 3 Faculté de médecine, Hôpital Cochin, Université Paris Descartes, 24 rue du Faubourg St Jacques, Paris, 75014, France; 4 UMR 1061, Unité de Génétique Moléculaire Animale, INRA/Université de Limoges, Université de Limoges, 123 Av. Albert Thomas, Limoges, 87060, France; 5 Unité de Génétique des Mammifères, Institut Pasteur, 25 rue du Docteur Roux, Paris, 75724, France; 6 Department of Animal Genetics, INRA, Domaine de Vilvert, Jouy-en-Josas, 78352, France

## Abstract

**Background:**

Accumulating evidence points to the mosaic nature of the mouse genome. However, little is known about the way the introgressed segments are regulated within the context of the recipient genetic background. To address this question, we have screened the testis transcriptome of interspecific recombinant congenic mouse strains (IRCSs) containing segments of *Mus spretus *origin at a homozygous state in a *Mus musculus *background.

**Results:**

Most genes (75%) were not transcriptionally modified either in the IRCSs or in the parent *M. spretus *mice, compared to *M. musculus*. The expression levels of most of the remaining transcripts were 'dictated' by either *M. musculus *transcription factors ('*trans*-driven'; 20%), or *M. spretus cis*-acting elements ('*cis*-driven'; 4%). Finally, 1% of transcripts were dysregulated following a *cis*-*trans *mismatch. We observed a higher sequence divergence between *M. spretus *and *M. musculus *promoters of strongly dysregulated genes than in promoters of similarly expressed genes.

**Conclusion:**

Our study indicates that it is possible to classify the molecular events leading to expressional alterations when a homozygous graft of foreign genome segments is made in an interspecific host genome. The inadequacy of transcription factors of this host genome to recognize the foreign targets was clearly the major path leading to dysregulation.

## Abstract 2

The testis transcriptome of mouse strains containing homozygous segments of *Mus spretus* origin in a *Mus musculus* background was analyzed.

## Background

Speciation is defined as the evolutionary process generating new species. It relies on reproductive isolation leading to the separate evolution of genomes. In the 'house mouse species complex' genomic exchanges do occur, and the laboratory mouse itself is considered as a mosaic of other subspecies. Indeed, laboratory mouse strains have originated from a limited number of founder populations of mixed genetic constitution [1,2].

A recent analysis of the fine structure of single nucleotide polymorphism (SNP) variation in the mouse genome revealed the existence of long segments with extremely high levels of polymorphism (one-third of the genome). This highly polymorphic subgenome is expected to originate partly from multiple subspecies [2], which suggests that the genomes of inbred strains (that is, *Mus musculus*) are mosaics of chromosome segments derived from other subspecies [1]. These results have been confirmed and extended to other mouse strains derived from the wild [3].

In spite of the accumulating evidence pointing to the mosaic nature of the inbred mouse genome in structural terms, little is known about the way the introgressed segments are regulated within the context of the recipient genetic background. Several microarray profiling experiments have been performed to compare expression in hybrid mice from different mouse subspecies and species [4,5]. In these studies, the target tissues were brain, liver and testes, as representative of behavior, metabolism and reproduction, respectively. The first study showed an excess in differentially regulated genes in the testis compared to brain and liver between *Mus spretus *and *M. musculus*. The second study completed the first by analyzing expression in the hybrid between subspecies; it confirmed the over-representation of genes differentially expressed in the testis compared to other tissues. Also, the authors suggest that inheritance is generally 'additive' (expression in the hybrid being generally near the midpoint between the expression of the two parent subspecies). Consistent observations have been independently published [6].

By contrast, other studies showed that hybrids may display 'non-additive' gene-expression patterns [7,8]. In the hybrid, the merging of two different subgenomes might lead to *cis*-*trans *incompatibilities that would explain the reported novel gene-expression patterns, as shown in *Drosophila *[9]. Indeed, it is expected that *cis*- (that is, regulatory sequences linked to the gene) and *trans*- (that is, transcription factors) regulatory elements within species coevolved through compensatory changes, and cannot always be mingled without so-called 'transcriptome shock', that is, massive gene dysregulation caused by the association of genomes that have evolved separately [10-12].

Most interspecific studies on gene expression profiling in mammals have been performed by analyzing separately the two or more species under scrutiny, or their hybrids. Clearly, expression in hybrids is made very complex, for instance, by the generation of a large quantity of abnormal heteromeric proteins [13]. Therefore, analyzing expression in a genuine mosaic genome would facilitate interpretation. Inter- or sub-specific hybrids constitute a first step in establishing a stable genomic mosaic, if followed by backcrosses and consecutive sib-pair crosses. In the present study, we took advantage of an original genetic model, a panel of interspecific recombinant congenic mouse strains (IRCSs) [14], to explore the behavior of chromosome segments introgressed in a foreign genome at a homozygous state. The model is composed of 53 strains obtained from interspecific crosses between C57BL/6 mice and the SEG strain derived from the species *M. spretus*. The C57Bl/6 genome is in fact composed of a mixture of unequal proportions of three distinct mouse lineages (*M. musculus domesticus*, *M. musculus musculus *and *M. musculus castaneus*) [2,15]. Despite the complexity of the species structure in mice, it is clear that *M. spretus *diverged from the house mouse complex more than 1.5 million years ago [16].

We show that the position of interspecifically introgressed segments is readily detectable by their expression alterations in the testis transcriptome. Using the IRCS model, we were able to classify the genes in categories according to their capability to correctly cope with the host genome due to their *trans*, *cis *or *cis *× *trans *dominant mode of regulation. In addition, we show that the gene expression dysregulation is correlated with the SNP content differentiating *M. musculus *and *M. spretus *in *cis*-regions.

## Results and discussion

### *M. spretus *segments in the IRCS are enriched in genes transcriptionally altered compared to B6

**Figure 1:**
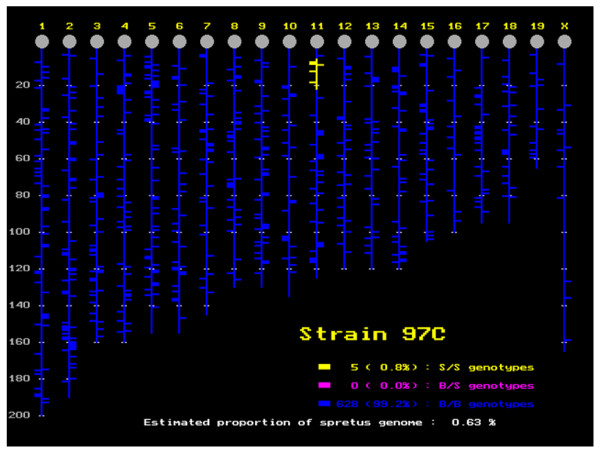
Position and size of the DNA segment of *M. spretus *origin in the *M. musculus *background for 97C, an IRCS used in the study. The segments of *M. spretus *origin are displayed in yellow. The small horizontal bars represent the position of genetic markers analyzed to build the map (see details in [14]). The picture was drawn before the analysis of testis expressional data, and a new segment found on chromosome 6 is thus not represented (see text); this segment is clearly visible on the expression profiles of chromosome 6 (Figure 6).

**Figure 2:**
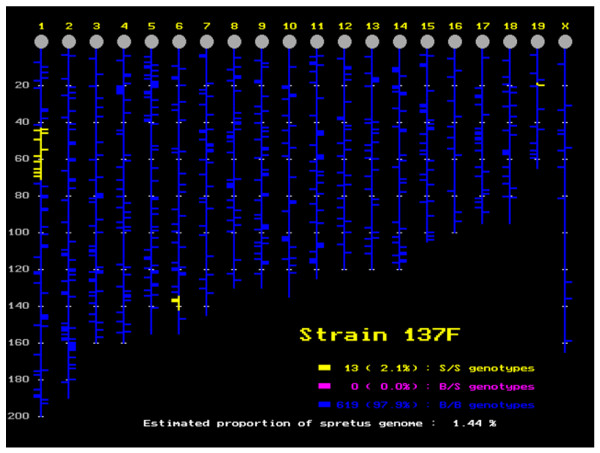
Position and size of the DNA segment of *M. spretus *origin in the *M. musculus *background for 137F mice. This strain contains three *M. spretus *segments estimated at 1.44% of the genome.

**Figure 3:**
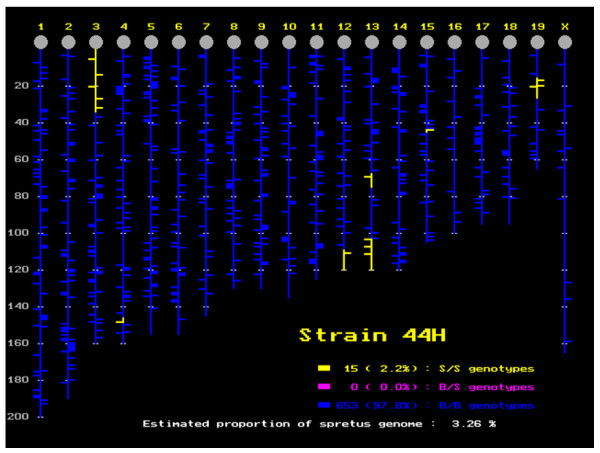
Position and size of the DNA segment of *M. spretus *origin in the *M. musculus *background for 44H mice, which apparently contains seven fragments. In fact, the small fragment on chromosome 15 was not confirmed, neither by the expression analysis nor by the genotyping of additional markers (see text, and Figures 5-7, green dots).

**Figure 4:**
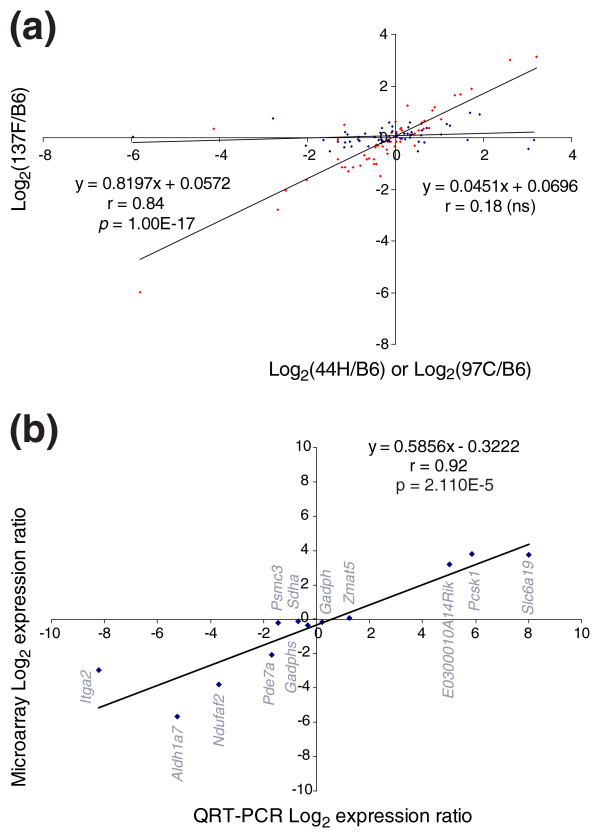
Assessment of reproducibility of the microarray data. **(a) **Linear regression analysis for the 60 genes located in an approximately 10 Mb region of *M. spretus *origin (MMU19) shared by 44H and 137F strains, and of *M. musculus *origin in the B6 and 97C strains (Figures 2, 3 and 7). The x-axis represents the log_2 _of the expression ratios between 137F and B6. The y-axis represents the log_2 _of the expression ratio between either 44H and B6, or 97C and B6. As expected, there is a highly significant correlation between the 44H/B6 ratio versus the 137F/B6 ratio (blue dots), since 44H and 137F both contain a segment of *M. spretus *origin at this chromosomal location, while there is no significant correlation between the 97C/B6 ratio versus the 137F/B6 ratio (red dots), due to dysregulations described in the text. **(b) **Linear regression between expression levels obtained by microarray or quantitative RT-PCR (QRT-PCR) for a sample of twelve genes.

In order to explore gene expression in a mosaic mouse genome, we have exploited existing IRCS mice. For this, we hybridized Nimblegen mouse expression microarrays with pooled testis cDNA (12 testes per strain) from three recombinant congenic strains (97C, 137F and 44H), and the parent strains C57Bl6/j (*M. musculus *B6) and SEG/Pas (*M. spretus*, SEG). Together, these three recombinant congenic strains carry about 5% of the introgressed *M. spretus *genome in a *M. musculus *background. Complete information on the strains, their origin, construction and mapping details is available in [14], and described in Figures 1, 2, 3 for the three strains under scrutiny. The Nimblegen arrays interrogate a total of 42,586 mouse transcripts, each transcript being represented by nine 60-mer oligonucleotides. We have found that the hybridization output is very robust. This translates into the fact that for 95% of the genome, we have four highly correlated fluorescence values per transcript (R > 0.98; see the Gene Expression Omnibus profile). This clearly shows that despite the genetic separation of the strains for more than 40 generations, their expression signatures are very similar. Indeed, this constitutes the most stringent criteria of biological replication. The same reasoning applies to the approximately 10 Mb *M. spretus *segment shared by 137F and 44H. Indeed, this constitutes a biological replicate for this *M. spretus *region (r = 0.84, *p *= 1.10^-17^, n = 59), while it drops to a non-significant value when the same fragment is compared between 97C (B6 genomic origin for this region) and 137F or 44H; this is illustrated in Figure 4a.

Moreover, we checked the microarray data by quantitative PCR and obtained a very good agreement (R = 0.92 (R^2 ^= 0.84), n = 12, *p *< 0.001; Figure 4b). We considered a transcript as expressed when the fluorescence level was >100, this fluorescence ranging from 20 to more than 60,000 (average approximately 3,100). The application of this threshold provided a selection of 37,432 transcripts (87.8%). To compare expression levels of transcripts between strains, we considered a gene as differentially expressed if a four-fold difference of expression was observed compared with the B6 parent. This threshold was chosen since it corresponds roughly to 1% of differentially regulated transcripts, a widely accepted threshold. The number of SEG and IRCS genes whose expression ratios with respect to B6 were modified at the four-fold threshold is summarized in Table 1. Between the two parent species, 20.9% of the transcripts were modified in the testis, with a similar amount of repressed and induced genes. Concerning the IRCS expression profiles, we found 0.09%, 0.18% and 0.23% of significantly modified transcripts at the pan-genomic level, for 97C, 137F and 44H, respectively. This is roughly proportional to the total size of *M. spretus *segments in each IRCS. Since this proportionality is lost for genes outside segments of *M. spretus *origin (that is, 'genetic background'), the correlation was mainly due to dysregulation of genes located within the *M. spretus*-derived segments. In 97C and 44H there was a significant excess of under-expressed genes, while interestingly, the opposite situation was observed in 137F. This could be due to the presence 'by chance' of one or a few potent transcriptional activators in the *M. spretus *segments of 137F.

**Table 1:** Dysregulated genes (compared to B6 expression levels) in *M. spretus *and the IRCSs

**All genes considered**				
Pan genomic (37,432)	SEG	44H	137F	97C
Under-expressed	3,290	57	15	26
Over-expressed	3,560	19	45	2
∑	6,850	76	60	28
Percentage of modified genes	20.90	0.23	0.18	0.09
Proportion of under-expressed (%)	48.0	75.0	25.0	92.9
				
**Only genes inside B6 segments considered**				
Genetic background (variable number according to the strain)		44H	137F	97C
Under-expressed		21	2	11
Over-expressed		3	30	0
∑		24	32	11
Percentage of modified genes		0.06	0.09	0.03
Proportion of under-expressed (%)		87.5	6.3	100.0

A statistical overview of genes with modified expression in the various genomic contexts examined in this study, compared to the B6 strain taken as a reference.

**Figure 5:**
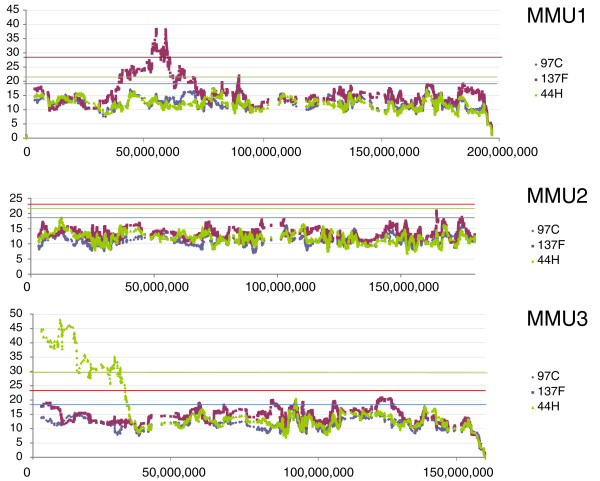
A representation of expression levels along IRCS chromosomes where *M. spretus *segments were detected by analyzing the testis transcriptome for chromosomes 1, 2 and 3. Chromosome 2 is represented as a negative control (no *M. spretus *segment). The graphs display the chromosomal position (abscissa) against the number corresponding to the sum of the log_2 _of the IRCS/B6 expression ratios in sliding windows of 50 genes (see Materials and methods). The horizontal lines represent a 1% probability of random occurrence estimated by one thousand random permutations of gene order, for each strain and each chromosome.

**Figure 6:**
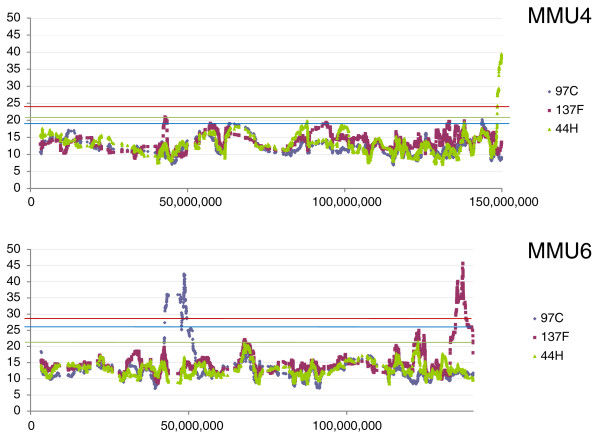
Sliding window representation of expression levels for chromosomes 4 and 6. The blue peak observed on chromosome 6 was not previously detected by genetic mapping. The existence of the segment was confirmed by genotyping new markers (see text).

**Figure 7:**
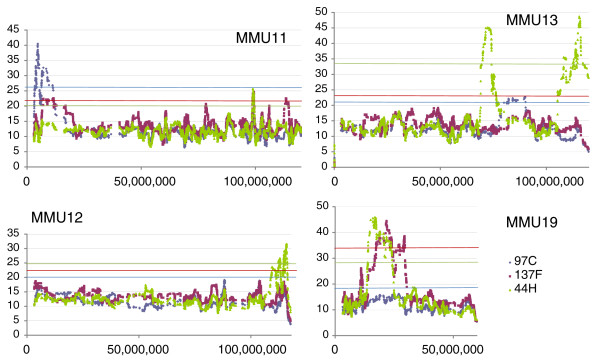
Sliding window representation of expression levels for chromosomes 11, 12, 13 and 19. Note the existence of a chromosome 19 fragment shared by 44H and 137F. This *M. spretus *region is regulated in a very similar way in both strains (Figure 5). Note that the size of the segment is apparently much larger than suggested by genotyping in 137F.

Next, we asked whether the *M. spretus *segments were homogenous in terms of gene expression dysregulation. In addition, we wished to test if dysregulated genes outside the *M. spretus *segments were clustered. We therefore determined the sum of the log_2 _of the expression ratios of induced or repressed genes in sliding windows of 50 transcripts (Figures 5, 6, 7). In order to test whether the number of modified genes in a given chromosome region was significantly higher than the background, we performed 1,000 permutations of gene order for each chromosome; for each of them the maximum value obtained was kept as a threshold for significance, represented by horizontal lines in Figures 5, 6, 7.

We could detect 11 clusters of modified genes. Ten of these clusters matched to IRCS segments according to available mapping information based on the genotyping of approximately 800 microsatellite markers and SNPs. Two discrepancies were observed, on chromosomes 6 and 15. On chromosome 6 (Figure 6), we detected a set of modified genes significantly clustered, which did not correspond to a known *M. spretus *segment in the 97C IRCS. To test whether this group was a region of B6 or SEG origin, we genotyped polymorphic microsatellites located inside the region (D6MIT224, D6MIT321, D6MIT313), which demonstrated the existence of a previously undetected approximately 9 Mb MMU6 *M. spretus *segment. By contrast, we failed to detect a small segment predicted by genotyping on MMU15 previously detected by a single SNP. Typing new microsatellites (D15MIT87 and D15MIT154, located at 0.6 and 0.2 Mb on each side of this SNP, respectively), did not make it possible to confirm the existence of a segment of *M. spretus *origin.

In short, we show that statistically assessing gene expression alterations made it possible to detect all *M. spretus *segments, uncover a previously undetected one (on MMU6), and disqualify a *M. spretus *segment that was very likely a false-positive (on MMU15). In the rest of the study, we will consider the combination of the 11 segments of *M. spretus *origin present in the three IRCSs as a whole. Overall, the proportion of dysregulated genes in the *M. spretus *segments inside the IRCS was 6.2% (144/2320) compared to 0.06% in the rest of the genome. Thus, the ratio of dysregulated genes is considerably higher in the *M. spretus *fragments than outside them.

**Figure 8:**
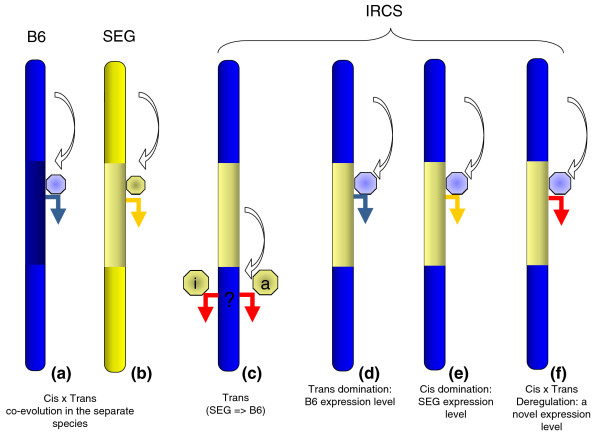
A summary of the modes of regulation encountered in the IRCSs and their parent strains. Blue marks the B6 chromosome segments while yellow marks the SEG chromosome segments. The blue arrows represent a B6 expression level, while the yellow arrows represent a SEG expression level. Red arrows correspond to expression levels that are different from the parent strains. The white arrows symbolize the interaction of *trans*-factors (octagons) on *cis*-regulatory elements, themselves located either on the same or on other chromosomes. **(a, b) **Parent strains B6 and SEG. **(c) **Representation of the interaction of an SEG *trans*-factor acting on the B6 genome. In the IRCSs, since the *M. spretus *segments account for less than 2% of the genome, this case is supposed to occur relatively rarely, but can probably explain the dysregulation of genes located in the B6 background. This dysregulation can be mediated either by an activating or inhibiting factor, the effect of these factors being detectable if the expression level is different from B6. **(d) **Interaction of a *trans*-factor of B6 origin on a *M. spretus *segment, resulting in a B6-like expression level. This type of regulation includes genes of class 1 (Figure 9). **(e) **Interaction of a *trans*-factor of B6 origin on a *M. spretus *segment, resulting in a SEG-like expression level (that is, the expression level is dictated by the *cis*-elements of *M. spretus *origin). This type of regulation includes genes of class 3 (Figures 9 and 10). **(f) **Interaction of a *trans*-factor of B6 origin on a *M. spretus *segment, resulting in a novel expression level. This type of regulation includes genes of class 2 (Figure 9).

We did not detect chromosome clusters of dysregulated genes outside *M. spretus *segments; however, we considered the possibility that these genes could belong to common functional pathways (functionally clustered). We analyzed induced and repressed genes in the genomic background of the IRCSs, using the DAVID functional classification tool to identify putative functional clusters [17]. This approach did not result in relevant and significant grouping of genes according either to a given function or to a specific keyword. This is likely due to the presence of more than one *M. spretus*-type transcription factor in the segments, each of them impacting on a restricted number of targets, which weakens the power of the clustering analysis. We observed that amongst the genes that are dysregulated in the *M. musculus *background of the IRCSs (67), about 90% (59) were also differentially regulated compared to their *M. spretus *orthologs. This suggests that transcription factors of *M. spretus *origin were unable to regulate *M. musculus *genes, neither in a B6 nor in a SEG fashion (Figure 8c).

### Exploring gene expression patterns in the IRCSs

In order to analyze more precisely the way genes are regulated in *M. spretus *segments, we calculated correlations between gene expression levels in the IRCSs and each of the two parent species, B6 (*M. musculus*) and SEG (*M. spretus*), and between the two parent species themselves. This analysis was carried out considering either the complete set of 37,432 transcripts, the restricted set of 2,320 transcripts located inside the segments (dysregulated or not), or exclusively the 144 modified genes (Tables 2 and 3).

**Table 2:** Total correlations in expression levels for the different mice of this study

	Correlation coefficients between expression levels					
	
	All transcripts (37,432)		All transcripts in the 11 segments (2,320)		Modifed transcripts in the *M. spretus *segments (144)	
			
	Correlation coefficient	*p*-value	Correlation coefficient	*p*-value	Correlation coefficient	*p*-value
B6 versus IRCSs	0.9905	<0.0001	0.9646	<0.0001	0.6899	<0.0001
SEG versus IRCSs	0.8846	<0.0001	0.9038	<0.0001	0.5963	<0.0001
B6 versus SEG	0.8921	<0.0001	0.8919	<0.0001	0.3243	0.002

Total correlations for the fluorescence levels of the various transcripts between the two parent species and the IRCSs taken as a whole. Three classes are considered, either with all the genes detectable on the array, or only the genes present inside the *M. spretus *segments, or only genes modified at the 4X threshold inside these segments. While even inside the *M. spretus *segments most genes are not dysregulated, a class of genes that are strictly driven by the *M. spretus *context appears, since the correlation between the IRCSs and SEG does not significantly decrease when the B6 parental effect is removed (from 0.5963 to 0.5441; see Table 3).

**Table 3:** Partial correlations in expression levels for the different mice of this study

	Correlation coefficients between expression levels, corrected for reciprocal parental effect					
	
	All transcripts (37,431)		All transcripts in the 11 segments (2,319)		Modifed transcripts in the *M. spretus *segments (143)	
			
	Correlation coefficient	*p*-value	Correlation coefficient	*p*-value	Correlation coefficient	*p*-value
B6 versus IRCSs	0.9561	<0.0001	0.8189	<0.0001	-0.1499	NS
SEG versus IRCSs	0.0155	NS	0.3639	<0.0001	0.5441	<0.0001

Partial correlations for the fluorescence levels of the various transcripts between the two parental species and the IRCSs taken as a whole. Three classes are considered, either with all the genes detectable on the array, or only the genes present inside the *M. spretus *segments, or only genes modified at the 4X threshold inside these segments. While even inside the *M. spretus *segments, most genes are not dysregulated, a class of genes that are strictly driven by the *M. spretus *context appears, since the correlation between the IRCSs and SEG does not significantly decrease when the B6 parental effect is removed (from 0.5963 to 0.5441; see Table 2). NS, not significant.

We found a strong positive correlation between B6 and SEG testicular transcriptomes (0.89, *p *< 0.0001), indicating overall a similar regulation of testis transcription in the two species. In a similar way, in the larger context of mammalian genome expression regulation, and expression regulation in various organs, a vast majority of genes is consistently regulated between species, such as human and chimpanzee [18], or cattle and pigs [19]. When the correlation was calculated between the IRCSs and B6, we observed a stronger correlation coefficient (0.99), indicating that introgressing about 2% of a foreign genome does not notably perturb the whole transcription profile, at least in the testis. We can therefore hypothesize that there is no strong detectable transcriptional epistatic effect between the introgressed segments and the genetic *M. musculus *background. Consistently, the correlation between the IRCS and SEG transcriptomes is very similar to that between B6 and SEG. Since this correlation may result either from a direct correlation between IRCS and SEG testicular gene expression or through a correlation involving the B6 genome, we also calculated partial correlations corrected for B6 effects (Table 3). This type of correlation is aimed at finding correlations between two variables after removing the effects of a third one. We observed that, on the whole transcript dataset, we lose completely the correlation between the IRCSs and the SEG parent (correlation coefficient shifts from 0.88 to 0.016), showing that this correlation between SEG and the IRCSs is indirect and can be almost entirely explained by their correlation with B6. It means that when the B6 testis transcriptome (taken as a reference) is removed from the analysis, no specific correlation persists between SEG and the IRCSs. This suggests that the same set of genes drives normal testis function in B6, SEG and the IRCSs.

Then, considering only the genes located in the 11 *M. spretus *segments of the IRCSs, we observed similar correlations to those observed at the pan-genomic level between B6 and the IRCSs, B6 and SEG, and SEG and the IRCSs (Tables 2 and 3). When partial correlations were considered, we conserved a significant correlation between B6 and the IRCSs and, interestingly, the correlation between the IRCSs and SEG became significant compared to the correlation computed using the whole set of transcripts (0.36, *p *< 0.0001 versus 0.016). This indicates that part of the genes located within the *M. spretus *segments conserve a SEG behavior even though they are present in a *M. musculus *background. This partial correlation between SEG and the IRCSs could, therefore, be a measure of the proportion of genes for which the regulation is independent from the genetic background. The correlation coefficient remained high between B6 and the IRCSs for genes located inside the *M. spretus *segment (0.82), showing that a majority of these genes is adequately regulated when introgressed in a background evolving separately for two million years [16]. This correlation can be taken as a measure of conservation of *cis*-*trans *co-evolution mechanisms, since it implies that B6 transcriptional factors are generally able to correctly regulate the expression levels of *M. spretus *genes driven by their original regulatory sequences.

We then calculated correlation coefficients for modified genes in the IRCS segments. The correlation was estimated at 0.69 between B6 and the IRCSs, which is still significant but, as expected, lower than in the pangenomic (0.99) or all-segments categories (0.96). This positive and strong correlation indicates that the observed dysregulation involves subtle quantitative effects, generally enhancing or decreasing gene expression without drastic inversion. Interestingly, when the correlation between B6 and the IRCSs is corrected for SEG effects, the correlation coefficient is not significant (-0.15), suggesting that the correlation essentially originates from genes regulated similarly between B6 and SEG in this category. Reciprocally, when the partial correlation is considered between the IRCSs and SEG for the same genes, we observed a positive and significant correlation coefficient (0.54), suggesting that, in this case, an important part of the dysregulated genes in the IRCS segments behaved in a SEG-like fashion.

In conclusion, when interspecific segments are introgressed, genes deal with the genomic environment according to various schemes: about 90% conserve their regulation; and approximately 10% are dysregulated compared to B6, but in this case most present an expression level consistent with the SEG parent.

**Figure 9:**
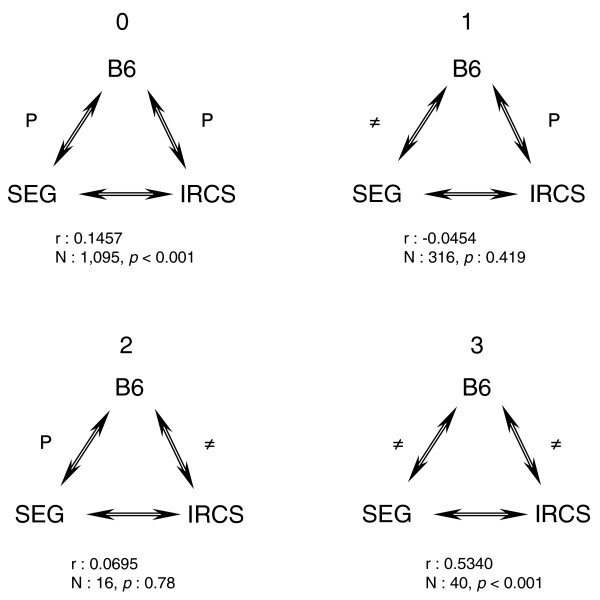
Triangles of correlations between the three groups of animals analyzed in the study. The analysis was carried out on the genes that were located in the *M. spretus *segments of the IRCSs, considered as a whole (n = 1,467). Four categories appear: genes that are consistently regulated in the parents and the IRCSs (class 0); genes that are differentially regulated between the two parents but regulated like B6 in the IRCSs (class 1); genes that are differentially regulated between the two parents but regulated in a SEG-like fashion in the IRCSs (class 3) - in this case the dysregulation between B6 and SEG on the one hand and B6 and the IRCSs on the other hand is associated with a strong correlation between gene expression in SEG and the IRCSs, meaning that the deregulation compared to B6 is not random and *cis*-driven; and genes that are similarly regulated between the parents but dysregulated (generally turned off) in the IRCSs (class 2).

In order to refine these conclusions, genes located within the *M. spretus *segments of the IRCSs (2,320) were categorized into four groups according to their expressional statuses relative to B6. First, we decided to filter genes modified between 2- and 4-fold from the dataset, which made it possible to keep 1,467 transcripts (Figure 9). The four classes were: class 0 (n = 1,095), genes that are not transcriptionally modified, neither in the IRCSs (*M. spretus *segments in a *M. musculus *genome), nor in the parental *M. spretus *mice (*M. spretus *segments in a *M. spretus *genome); class 1 (n = 316), genes that are not modified in the IRCSs, but are modified in *M. spretus*; class 2 (n = 16), genes that are modified in the IRCSs but not in *M. spretus*; and class 3 (n = 40), genes that are modified both in the IRCSs and in *M. spretus*. We also calculated correlation coefficients between the IRCSs and SEG for genes in each class after correction for the B6 effect (partial correlations; Figure 9).

For genes belonging to class 0, the expression behavior is compatible with two non-exclusive possibilities: low *cis*/*trans *divergence; and, in presence of genuine *cis*/*trans *divergence, the robustness of the transcriptional response buffers these variations.

The expression of genes of class 1 was not correlated between the IRCSs and SEG. This suggests that, in this case, *trans*-acting factors from the B6 background are able to bring gene expression to a B6-like level (*trans*-driven effect). Taking into account that this category is abundantly represented (about 20% of genes in the segments), these differences between B6 and SEG could be relevant for understanding species-specific differences in gene expression.

For genes of class 2, the least abundant class (about 1%), there was no correlation between SEG and the IRCSs. Since these genes were regulated similarly between SEG and B6, we concluded that their expression was disrupted in a new fashion, due to their introgression in an interspecific genetic background. It can be hypothesized that selection acted to maintain a constant level of gene expression through time (and so maintain a phenotype) by developing compensatory *cis *and *trans *changes (*cis*/*trans *co-adaptation). In consequence, for genes of class 2, when SEG segments are introgressed in the B6 genome, *cis*- and *trans*-regulatory elements are no longer adjusted and gene expression is dysregulated. Similar observations based on theoretical and experimental considerations have been published recently [9,20].

**Figure 10:**
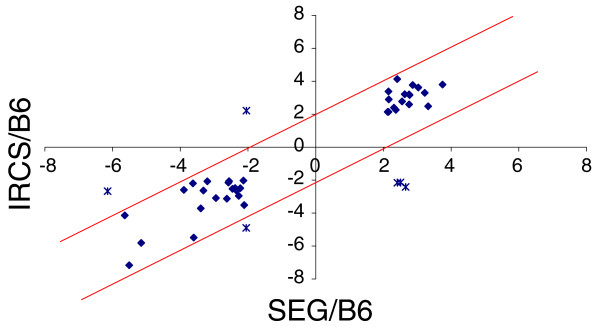
Graph representing the expression of class 3 genes in the IRCSs compared with SEG, with B6 expression taken as a reference. Specifically, we plotted the expression ratio IRCS/B6 against SEG/B6. The two lines define a four-fold variation threshold for the expression ratios. Most genes belonged to the interval defined by the two lines. The expression of these genes was presumably driven to a SEG expression level by their *cis*-elements of SEG origin. Only six genes were really dysregulated (represented by asterisks instead of diamonds).

For genes belonging to class 3 (dysregulated in the IRCSs and presenting a different expression level between B6 and SEG), we found a significant positive correlation between the IRCSs and SEG (r = 0.53, *p *< 0.0001). This indicates that these genes, regulated differently between the two parental species, and differently expressed in the IRCSs compared to B6, keep a SEG behavior in a B6 background. The regulation of these genes is probably *cis*-driven, suggesting that their proximal regulatory elements are sufficient to yield a *M. spretus*-like expression level, whatever the background. It was interesting to check whether all these genes behaved in a similar way between SEG and the IRCSs. Therefore, we plotted the transcriptional log ratio of the IRCSs/B6 versus SEG/B6 (Figure 10). This graph shows that, amongst the 40 genes of class 3, 6 did not display a SEG-like expression level. Four genes were even regulated in an opposite fashion. These outliers explain why the correlation coefficient was only of 0.53.

We also tried to characterize these expression classes at the functional level, using the DAVID software, but we did not succeed in clustering genes in functional groups or according to specific keywords.

**Figure 11:**
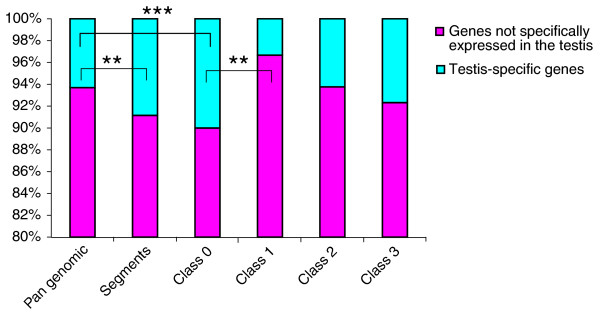
The proportion of testis-specific genes in the different subgenomes studied. Significantly different values are represented by horizontal brackets (at least significant at *p *< 0.01). On average, the segments are enriched in testis-specific genes, essentially owing to the bulk of genes from class 0 (75%). By contrast, class 1 is depleted in testis-specific genes, suggesting that *trans*-driven dysregulation (forcing *M. spretus *genes to a B6 behavior) is strongly counter-selected. The increased number of testis-specific genes in the segments could be due to a positive selection aiming at compensating deleterious effects on reproductive parameters during the process of strain establishment. **p-value < 0.01; ***p-value < 0.001.

We then evaluated the testis-specific gene proportion in each class, at a pan-genomic level and inside *M. spretus *segments (Figure 11). At the 'pan-genomic' level, approximately 6% of the genes were specifically expressed in the testis. This value was significantly different from the proportion of testis-specific transcripts in the IRCS segments (8.8%, *p *< 0.0002), as well as from the percentage of testis-specific genes of class 0 exclusively (10.0%, *p *< 0.0002). This indicates that *M. spretus *segments are enriched in testis-specific genes belonging to class 0 (genes from *M. spretus *segments that are regulated similarly, either in the B6 or SEG genomic background). This enrichment could be due to the selection of non-dysregulated genes that may be relevant for testis function. When the different classes of genes were compared with respect to their testis-specific gene content, only classes 0 and 1 (encompassing only 4% of testis-specific genes) were significantly different (*p *< 0.0019). This suggests that genes differentially expressed between SEG and B6, owing to differences in *trans*-driven regulation, may be less prone to be testis-specific.

### Expression alterations are associated with a high number of SNPs in the promoter of genes located in the *M. spretus *fragments

We wished to test whether differences in gene expression between SEG and B6 were due to promoter evolutionary divergence. For this, we amplified and sequenced the *M. spretus *proximal promoters of 24 genes located inside IRCS *M. spretus *fragments (500-1,500 bp upstream of the ATG, based upon the outputs from the Genomatix portal [21]. We compared a set of 19 promoters of genes modified at the expression level, irrespective of whether they were over-expressed or down-regulated, with a set of 5 promoters corresponding to genes with unaltered expression (Table 4). The 19 promoters belonged to classes 2 (4 genes) and 3 (15 genes). The five promoters corresponding to genes with unaltered expression were from class 0, considered as the best possible control for non-varying transcripts. Overall, we estimated the *M. spretus*/*M. musculus *sequence divergence at 2.7% in the promoters of dysregulated genes, versus 1.1% in the promoters of unmodified genes. The number of differences, either absolute or corrected for sequence length, were significantly different (*p *= 0.008 or 0.016, respectively). Genomatix was then used to compare the transcription factor binding site (TFBS) content between the B6 and SEG versions of the 24 promoters. We calculated the number of differences in TFBS content between the two versions of each promoter, in absolute terms and relative to the total number of TFBSs. We detected significant differences between the two groups of promoters (27.2 versus 12.2, *p *= 0.019, and 21.6% versus 10.5%, *p *= 0.023, respectively). For each of these parameters, we did not observe significant differences between over-expressed and down-regulated genes; similarly, no difference was visible between genes from classes 2 and 3.

**Table 4:** Comparisons between *M. spretus *and *M. musculus *promoters

Gene name	Induction ratio IRCS/B6	Length of B6 promoter (bp)	Length of SEG promoter (bp)	Number of sequence differences	Percentage of sequence divergence	Total number of TFBSs detected with Genomatix	Number of TFBSs differing between *M. spretus *and *M. musculus*	Percentage of TFBS differing between *M. spretus *and *M. musculus*
**Genes with modified mRNA level between SEG and B6**								
Pde7a	0.02	749	728	29	3.87	137	21	15.33
Aldh1a7	0.04	598	587	11	1.84	101	30	29.70
1810058I14rik	0.05	638	613	28	4.39	100	38	38.00
Plcz1	0.09	1,432	1,392	42	2.93	228	51	22.37
Itga2	0.10	769	749	21	2.73	94	14	14.89
Nol9	0.11	1,034	1,021	15	1.45	198	32	16.16
4833412L08Rik	0.12	601	583	18	3.00	106	20	18.87
4921521F21Rik	0.16	603	597	7	1.16	137	8	5.84
Plek	4.34	698	679	27	3.87	134	33	24.63
Cast	5.28	648	629	21	3.24	101	22	21.78
Slc6a3	5.94	657	650	7	1.07	90	16	17.78
Hnf4g	6.64	601	589	14	2.33	105	29	27.62
6130401L20Rik	7.22	758	743	26	3.43	138	53	38.41
Mbl2	7.45	629	615	14	2.23	101	24	23.76
E030010A14	10.06	622	600	33	5.31	143	63	44.06
Hebp1	10.19	604	596	9	1.49	83	11	13.25
Pcsk1	20.74	839	834	5	0.60	110	9	8.18
Slc6a19	23.39	667	648	22	3.30	96	22	22.92
Pdcd1lg2	38.40	662	647	17	2.57	88	20	22.73
**Mean**		**726.8**	**710.5**	**19.3**	**2.7**	**120.5**	**27.2**	**21.6**
**Standard deviation**		**201.9**	**197.4**	**9.9**	**1.2**	**37.9**	**15.2**	**11.1**
								
**Genes with unchanged mRNA level between SEG and B6**								
Tle4	1.19	1,289	1,284	5	0.39	250	5	2.00
Bhlhb5	1.20	791	788	3	0.38	165	11	6.67
Cypt12	1.07	603	593	12	1.99	161	14	8.70
Spag16	0.81	511	506	6	1.17	87	13	14.94
Zmat5	0.94	619	610	9	1.45	90	18	20.00
**Mean**		**762.6**	**756.2**	**7.0**	**1.1**	**150.6**	**12.2**	**10.5**
**Standard deviation**		**311.2**	**312.3**	**3.5**	**0.7**	**66.9**	**4.8**	**7.1**
***p*-value**		**NS**	**NS**	**0.008**	**0.016**	**NS**	**0.019**	**0.023**

Sequence and comparative analysis of putative TFBSs between *M. musculus *and *M. spretus*. Genes with unchanged mRNA levels between SEG and B6 consist of *M. spretus *genes that were not dysregulated when introgressed in the B6 genome. We observed (see text) that modified genes present half as much sequence variation and difference in putative TFBSs. Statistical values (mean and standard deviations) are represented in bold, as well as the *p*-values showing statistically significant differences in sequence divergence and TFBSs between modified and unmodified genes (Mann-Whitney non-parametric test). NS, not significant.

These data suggest a mechanism explaining the difference in gene expression characterizing the *M. spretus *fragments in a B6 context. These may be due to differences in the promoter sequences that alter their interaction with the relevant transcription factor(s), either due to the modification of the binding sequence, or the number of binding sites, or abolishing any possible interaction.

## Conclusion

Up to now, most transcriptional studies aiming at understanding interactions between different genomes have been carried out using inter- or intersub-specific hybrid animals [5,9,12,22] and polyploid and hybrid plants [10,23,24]. In the case of *M. musculus*, it is known that the genome harbors segments from various subspecies (*M. musculus domesticus*, *M. musculus molossinus*, *M. musculus musculus*) [1,3]. In the present study, we used an original mouse model to explore genome-wide gene expression, in a context of interspecific mosaicism. Specifically, homozygous segments of *M. spretus *origin were introgressed in a *M. musculus *background. *M. spretus *and *M. musculus *diverged about two million years ago, accumulating an interspecific divergence estimated at 1% [16,25].

The process to obtain the IRCSs involved an interspecific cross followed by two backcrosses on a *M. musculus *background and finally consanguineous crosses over more than 20 generations. As a result of this process, it was expected an average of about 12.5% of *M. spretus *material introgressed within the final IRCS genomes. However, the actual proportion is currently estimated at about 1.37% (range 0-3.79%, according to detection with approximately 800 polymorphic DNA markers). This observation, together with the fact that during the process of strain establishment 55% of the strains did not survive, indicates that there was strong selection acting against the maintenance of the *M. spretus *fragments in the *M. musculus *background. Such a counter-selection would be consistent with the 'Muller-Dobzhansky' model, proposing the existence of deleterious interactions between genes that have evolved in separate populations, which constitutes the genetic basis for speciation [26]. In molecular terms, it is now acknowledged that 'genomic shock' occurs in various interspecific hybrids [27,28]; thus, in the present day IRCSs, the retained segments are expected to be the least deleterious fraction of *M. spretus *chromosomes that could go through the interspecific barrier.

Despite the fact that about 50% of the initial strains survived, we have shown in a previous study that they are often hypofertile, and display various non-lethal anomalies of the male function and genital tract (small testes, teratozoospermia, partial Sertoli-cell-only phenotype, abnormalities in the development and function of annex glands) [29]. We hypothesize that genes dysregulated exclusively in the IRCSs (defined as class 2 in the present study) are the basis of the molecular alterations leading to reproductive defects. We ruled out the hypothesis that the expression alterations could be due to variations in the relative percentage of testis cell types in the different strains. Indeed, this has been checked by histology [29]. In addition, such modification would induce gene expression modifications in all the genome (including the pure B6 genetic background), which was not observed. Even in the 137F strain where approximately 10% of seminiferous tubules are without germ cells, such an alteration was not observed. More specifically, the expression of genes that mark specific testis cell types (such as *Ar*, *Amh *for the Sertoli cells, *Cyp17a1 *or *Hsd17b1 *for Leydig cells) and meiosis genes for germ cells (such as *Spo11*, *Sycp3*) or spermatogenesis specific genes (such as *Prm1 *and *2 *or *H1t*) was not altered in the B6 background of the strains.

We observed that introgressed segments were enriched in testis-specific genes of class 0 (10.0%, versus 6.3% for the whole genome). This suggests that despite selection against interspecific segments, the introgression of genes potentially important for reproduction do occur, provided that they undergo a similar regulation as in the two parent species. By contrast, class 1 (that is, encompassing genes behaving like B6 in the IRCSs) contains less testis-specific genes (<4%), indicating that a selection process might have acted against their retention. As a result of this selective stringency, about 95% of the introgressed genes are correctly regulated relative to their *M. musculus *orthologs, either because *M. spretus *and *M. musculus *regulation is similar, or because the *M. musculus trans*-factors govern and determine the expression level (class 0 and 1, respectively).

Interestingly, Rottscheidt and colleagues [5] showed that, in the case of interspecific hybrids, the vast majority of testicular transcripts were expressed at an intermediate level between the two parents (a property called 'additivity' in their study). This was different in the cross *M. m. domesticus *× *M. m. castaneus*, which was the most divergent one (approximately one million years ([5] and references therein), for which a similar proportion of 'additively' and 'non-additively' expressed transcripts was found. In the present work, we observed that within *M. spretus *segments in the IRCSs, approximately 95% of the transcripts showed a *M. musculus*-like expression level (belonging to classes 0 and 1). For such genes of class 0, the minimal hypothesis is a satisfactory *cis*/*trans *match between SEG and B6, resulting in 'additive' expression in the Rottscheidt sense. In addition, for genes of class 1, B6 transcription factor(s) dictate the expression level, and force it to a B6-like level (according to the Rottscheidt definition, this case is not additive; we could call it 'B6-dominant'). Only approximately 3% of the genes presented a *M. spretus *expression level in the IRCSs (genes belonging to class 3): in this case, the *M. spretus cis*-element dictates a *M. spretus*-like expression level (according to the Rottscheidt definition, this behavior is 'SEG-dominant'). Less than 1% of genes had an expression level in the IRCSs that was significantly different from that of both parent species (class 2). In this case, the Rottscheidt definition of additivity or dominance does not fit. In this category, we observed that about 80% of the transcripts were under-expressed (often close to extinction) compared to both B6 and SEG, indicating that *cis*- and *trans*-elements do not match.

This relative abundance of genes presenting a 'non-additive' behavior might be explained by the fact that *M. musculus *and *M. spretus *split approximately two million years ago (and are therefore more divergent than the most divergent cross performed in the Rottscheidt study). Our study is in fact different, since we observed effects for the comparison between *M. musculus *and a species outside the 'house mouse species complex'. Alternatively, and not exclusively, the relative importance in 'non-additive' gene expression could also be explained by the other peculiarity of our model: the homozygosity of the *M. spretus *segments, which obliges *trans*-regulators of one species to act on *cis*-elements of another species. In the hybrid, by contrast, the co-existence of two 'hemigenomes' allows *trans*-factors to find their *bona fide *targets at least in the 'right hemigenome'.

The relative simplicity of our model makes it possible to decipher and classify regulation incompatibilities between *M. musculus *and *M. spretus*, contrasting the effect of *trans*-driven regulation (class 1), *cis*-driven regulation (class 3) and *cis × trans *mismatches (class 2) (Figure 8). The genomic incompatibilities that we observed are to be placed in an evolutionary context in the establishment of an inter- or intersub-specific mosaic genome. It is well-known that gene flow across species is limited in the first generation by several hybrid sterility loci, which constitutes a first barrier against interspecific hybridization (for recent work, see [30]). Interestingly, Oka and coworkers showed that fertility continues to drop in successive backcrosses, following an interspecific cross [31]. In this context, it is possible to map quantitative trait loci (QTL) involved in this secondary frontier against interspecific mingling, this phenomenon being called hybrid breakdown. We observed that this decreased reproductive fitness is still present when introgressed fragments are stabilized in a mosaic genome. Such a phenomenon probably maintains the fraction of introgressed interspecific genome relatively low in IRCSs and also probably in natural populations. The proportion and the quality of this retained fraction have been measured across a hybrid zone between *M. m. domesticus *and *M. m. musculus *[32]. The authors found genome segments spreading from one species to the other, containing in some cases genes involved in the reproductive process. In a recent study based on a *M. musculus *× *M. domesticus *cross aiming at establishing consomic strains, this phenomenon was encountered again [33,34].

In our previous study [29], we showed that these incompatibilities can readily be used to map QTL involved in male fertility parameters. IRCSs are a very powerful tool to dissect the genetic bases of various phenotypes since *M. spretus *segments introgressed in the *M. musculus *genome ensure access to regions that were up to now 'blind' (non-polymorphic) and, therefore, non-exploitable for QTL mapping [3]. From the point of view of physical mapping, the dysregulation that we observed can be used as a very powerful method to exhaustively detect introgressed segments. Indeed, even if only 10% of the genes are dysregulated, since approximately 45,000 transcripts are analyzed in the microarray, 4,500 expressional markers will be informative for mapping and, thus, ensure a very high resolution. Along with the IRCS set described in [14] and analyzed here, there are other similar tools [33,34] that might be exhaustively studied using the expression mapping approach outlined here.

## Materials and methods

### Mouse strains

The parent strains were *M. spretus *(SEG/Pas strain: SEG) and *M. musculus *(C57BL/6J strain: B6). To construct the IRCSs, F1 females (B6 × SEG) were crossed with B6 males. Fertile backcross males were mated with B6 females and their progeny were brother-sister mated for over 20 generations to produce inbred strains. At the time of the study, 43 out of the 53 strains investigated had more than 40 generations of inbreeding. The animals were bred in Pasteur's animal facility until weaning and then housed in a controlled environment (light/dark cycle, temperature, free access to mouse food and water) in the animal facility of the Cochin Institute. All mice were raised under identical standard laboratory conditions and were sacrificed at the age of 6-8 weeks. All the experimental procedures were conducted in accordance with the policies of the University and the Guidelines for Biomedical Research Involving Animals.

### RNA extraction

Total testis RNA was extracted using TRIzol Reagent (Invitrogen, Carlsbad, CA, USA) in accordance with the manufacturer's instructions. RNA extractions from the two testes of six males of each strain were pooled before DNase I treatment (Invitrogen, Carlsbad, CA, USA).

### Gene expression arrays

Twenty micrograms of RNA from each strain (B6, SEG, and the IRCSs 44H, 137F and 97C) were sent to the NimbleGen expression array platform (Nimblegen, Reykjavik, Iceland). cDNA syntheses, DNA end-labelling, hybridization, scanning, and data normalization were performed at the NimbleGen facility. Hybridizations were performed on the standard expression design for mouse (mm8; NCBI Build 36, *M. m. domesticus*) corresponding to 42,586 exemplar genes representing a total of 53,127 transcripts/variants with nine 60-mer probes per gene. Nimblegen provided the final normalized data files.

### Chromosome-wide expression level visualization

The average fluorescence values for each transcript were inserted in an Excel file, chromosome per chromosome for each strain analyzed. These fluorescence levels, considered as expression values, were divided gene per gene by the corresponding ones from B6, taken as a reference. The logarithm (base2) of these ratios was calculated. To evaluate gene modification along a portion of chromosome, the absolute values of these log_2 _(ratios) were calculated, and these values were summed using a sliding window comprising 50 genes. The results are presented in Figure 2.

### Correlations and partial correlations

Normalized fluorescence levels for each transcript of each strain were analyzed using SPSS software (V8.0.1F) (SPSS Inc., Chicago, IL, USA). Correlation and partial correlation coefficients, as well as *p*-values for significance were computed using SPSS.

### Testis specificity assignment

The dataset corresponding to the Mouse GNF1M (gcRMA-condensed) GeneAtlas of the SymAtlas website [35] was retrieved from the site [36]. We defined 'testis-specific' genes as genes with testis expression at least 3-fold above the median gene expression in the 63 tested tissues, and that were expressed in less than 3 tissues out of the 63 tested. Differences in the testis-specific gene proportion in each expression class (at the pan-genomic level, in the *M. spretus *segments or in the four classes 0, 1, 2 and 3) were tested using the 'significance of the difference between two independent proportions' function of VassarStat [37].

### *M. spretus *promoter sequencing

B6 proximal promoters corresponding to 500-1,500 bp upstream of the ATG were retrieved from the Genomatix portal [21] for 55 genes. PCR primers (primer sequences available upon request from the authors) were designed based upon the B6 sequence and used to sequence SEG promoter versions on pooled genomic DNA extracted from tail tissues from six animals. PCR was performed using dimethyl sulfoxide (DMSO) enhancer, and Platinum™ Taq DNA polymerase (Invitrogen). We obtained an amplification product for 24 promoters. PCR products were purified and sequenced. *M. spretus *sequences were blasted against the B6 promoter sequences, taken as reference. Differences in the number of putative TFBSs between orthologous promoters were identified using the Genomatix GEMS launcher task function. Sequence divergence, in absolute terms (percentage of similarity) or relative to the length of the corresponding promoters between B6 and SEG sequences were statistically tested using Mann-Whitney tests. A similar approach was used for estimating and statistically testing the number and differences in putative TFBSs. The accession numbers for *M. spretus *promoters, which have been obtained from GenBank, are available in Table S1 in Additional data file 1.

### Transcriptome data

The transcriptome data have been deposited at Array express (accession number E-TABM-444).

## Abbreviations

IRCS, interspecific recombinant congenic strain; QTL, quantitative trait locus; SNP, single nucleotide polymorphism; TFBS, transcription factor binding site.

## Authors' contributions

DLH performed the experiments, made most of the correlation analysis and contributed to the writing of the paper. CS performed the experiments and brought expertise in testis physiology. RAV contributed to the writing and editing of the manuscript. XM produced and supervised the breeding of the mouse strains. AO provided advice on the manuscript. DV conceived the experimental design, participated in the data analysis and interpretation, and contributed to the writing and editing of the manuscript. All authors read and approved the final manuscript.

## Additional data files

The following additional data are available. Additional data file 1 is a table listing accession numbers for *M. spretus *promoters obtained from GenBank.

## Supplementary Material

Additional data file 1 Accession numbers for *M. spretus *promoters obtained from GenBank. Click here for file
